# Infection control in child daycare centers: logistics, knowledge, and practices of caregivers

**DOI:** 10.1186/s42506-019-0016-7

**Published:** 2019-05-03

**Authors:** Mohamed Mostafa Tahoun, Ali Abdel Halim Hasab, Nessrin Ahmed El-Nimr

**Affiliations:** Department of Epidemiology, High Institute of Public Health, Alexandria University, 165 El-Horreya Ave. El-Hadara, Alexandria, 21561 Egypt

**Keywords:** Caregivers, Daycare centers, Infection control, Knowledge, Practice

## Abstract

**Background:**

Children attending daycare centers (DCCs) are at high risk of contracting infectious diseases due to several factors including lack of knowledge among the caregivers about infection prevention and control practices. The objectives were to describe the DCC features, infrastructure, and infection control logistics, to assess knowledge of DCC caregivers regarding infectious diseases, and to assess their infection control practices.

**Methods:**

Using a cross-sectional design, 402 caregivers working in 59 DCCs in three districts in Alexandria, Egypt were included. Data were collected using a data collection sheet about the DCC features, a structured interviewing questionnaire to collect data on caregivers’ personal characteristics, knowledge about infectious diseases, and the best infection control practices and an observational infection control practices checklist. Multiple analysis of variance was used to test the difference in two or more vectors of means (mean knowledge scores about infectious diseases and about infection control). Post hoc test using Tukey Honest Significant Difference was used to determine which groups in the sample differ. Regression analysis models were used to identify factors affecting knowledge score of caregivers, and to estimate the magnitude of the association between different variables and the level of practice of caregivers (poor/fair and good).

**Results:**

Satisfactory features of the DCCs included the aeration, level of cleanliness, and availability of hand washing facilities, while the availability of gloves and aprons, alcohol-based products, and medical examination rooms were not satisfactory. Only 2.5% of caregivers had a good level of knowledge. Level of education was the only factor statistically associated with the level of knowledge. About 31% and 17% had poor and good practice score percent, respectively. District and daily working hours were the only variables statistically associated with the level of practice.

**Conclusion:**

The level of knowledge and practice of caregivers was below optimum.

## Introduction

It has been accepted since the 1940s that there is a higher frequency of infectious diseases among children in collective out-of-home care [1]. Children attending daycare centers (DCCs) acquire infections, including respiratory infections, acute otitis media, diarrheal disease, invasive bacterial disease from *Haemophilus influenzae* and *Streptococcus pneumoniae*, hepatitis A and infections by CMV and varicella-zoster [2–4], more often than children cared for at home [2, 3, 5]. These infections can cause parental stress, secondary transmission, healthcare costs, and costs for parental work absence [6–8].

Studies have discussed the association between DCC attendance and the risk of infectious diseases among children. Children with congenital heart disease, chronic lung diseases, or other underlying diseases were at greater risk of severe infectious complications [9]. In Alexandria, Egypt, the prevalence rate of parasitic infections among children in DCCs in 1995 was 56.0% [10]. Possible transmission of HBV in DCCs was reported [11], while in the USA, no cases of HIV infection were known to have resulted from the transmission of the virus in DCCs. The risk of transmission of HIV by percutaneous body fluid exposure, such as biting was low [12].

Transmission of an infectious agent within a child DCC is influenced by several factors including the characteristics of the children attending (for example, age, sex, and immunological status), family characteristics, and length of time enrolled at the center [1]. DCC attendance has been associated with an increased risk for hospitalization for acute respiratory and gastrointestinal infections among children younger than 1 year of age [4]. DCCs environment also plays a role in the transmission of infectious agents [13, 14]. The size of the DCC is among these factors. Infection rates are generally higher at large DCCs. Other factors include the class size, structural design, quality of sanitary facilities, and infection control policies and practices followed in the facility [15].

In addition, caregivers themselves have an impact on the child’s health. The number of workers per child and their hygienic practices, in particular, on the hygiene involved in handling children play a role in the transmission of the infection within DCCs [1, 15]. A positive relationship has been established between hygienic training of caregivers and the reduction of illness [16].

Infection control standards are recommended for environments in which children are cared for together [17]. Infection control programs should be applied in the DCCs to reduce pediatric infections. The program should involve training of the caregivers on the following: hand washing, environmental cleaning, and disinfection (washing and disinfecting bathrooms and other surfaces, diaper changing areas, potty chairs and toilets, toys, and cleaning up body fluids), standard diaper changing steps, and food safety [18].

The objectives of the present study were to describe the DCC features, infrastructures, and infection control logistics in Alexandria, to assess the knowledge of the DCC caregivers about infectious diseases and their modes of transmission and to assess the infection control practices of the DCC caregivers.

## Methods

The study was conducted at the DCCs in Alexandria, Egypt using a cross-sectional design. The sample size was calculated using Epi Info 7.1.3.3 program (CDC, Atlanta). Based on the assumption of a prevalence of good knowledge among caregivers in DCCs of 50% and 5% confidence limit, the minimum required sample size at 95% confidence level was 384 caregivers. The sample was rounded to 400 and 402 caregivers were included. Each caregiver was observed 3 times consecutively with a total of 1206 observations.

According to the Directorate of Social Solidarity records (2013), Alexandria is divided into 6 districts with a total of 700 DCCs, and an average of 5 workers in each one of them (personal communication). Three districts were selected randomly, and caregivers were proportionately allocated according to the total number of caregivers in each selected district. A total of 59 DCCs licensed and supervised by the Ministry of Social Solidarity in Alexandria, Egypt were included and all caregivers in the chosen DCCs (teachers, nannies, kitchen workers, and cleaning workers) were also included (27 DCCs from district X with 208 caregivers, 12 DCCs from district Y with 75 caregivers and 20 DCCs from district Z with 119 caregivers).

Data was collected using a data collection sheet about the DCC features, a predesigned interviewing questionnaire with caregivers and an infection control checklist. The data collection sheet about the DCC features was filled by interviewing each of the DCCs directors, and by observing the DCC features, infrastructure and the infection control logistics. It included data about the nursery (design, location, number of children, number of classrooms, presence of playground, and source of drinking water), classrooms (aeration and using disinfectants in cleaning), bathroom (cleaning schedule and level of cleanliness), kitchen (level of cleanliness, food preservation method, and refrigerator cleanliness), and IC logistics (availability of soap and water, alcohol-based substances, personal protective equipment (PPE), diaper changing room, and its features if present).

A predesigned structured interviewing questionnaire was used to collect data from caregivers regarding their personal characteristics (age, sex, job, level of education, years of experience, marital state, infection control training, childcare training, number of children they are responsible for, and whether those children were located in one class or distributed in more than one class), knowledge regarding infectious diseases, modes of transmission, and the possible ways of infection prevention and control and knowledge regarding infection control practices. The total knowledge score was calculated by summing scores of all questions yielding a total score ranging from 0 to 50 and was classified into poor (< 50% or < 25 points), fair (50–< 75% or 25–< 38 points) and good or satisfactory level of knowledge (≥ 75% or ≥ 38 points).

Finally, an infection control checklist was used for observation of the following infection control practices: hand washing, use of PPE, waste disposal, diaper changing, environmental cleaning, and meals and feeds preparation. A special scoring system was constructed according to the job responsibilities of each caregiver. Accordingly, the practice score percent was calculated, and the percentage range was classified into poor (< 50%), fair (50–< 75%), and good or satisfactory levels of practice (≥ 75%).

### Statistical analysis

The collected data were coded, entered, and cleaned using SPSS for Windows version 21.0 (SPSS Inc., Chicago, IL, USA) and SAS for Windows version 9.4 (SAS Institute Inc., Cary, NC, USA). For quantitative variables, mean and standard deviation were calculated. Multiple analysis of variance (MANOVA) was used to test the difference in two or more vectors of means (mean knowledge scores about infectious diseases and about infection control). Post hoc test using Tukey HSD (Honest Significant Difference) was used after MANOVA to determine which groups in the sample differ. Multiple regression analysis was used to identify factors affecting knowledge score of caregivers, while stepwise logistic regression analysis was carried out to estimate the magnitude of the association between different variables and the level of practice of caregivers (poor/fair and good). A *p* value < 0.05 was considered to be statistically significant.

## Results

The number of classes per DCC ranged from 2 to 7 with a mean of 3.5 ± 1.1 classes (median = 3) and the number of children per class ranged from 1 to 23 with a mean of 12.1 ± 3.9 children (median = 11). The number of water sinks ranged from 1 to 11 sinks per DCC with a mean of 4.6 ± 2.0 sinks and the number of children per water sink ranged from 3 to 47 children per sink with a mean of 10.9 ± 7.6 children. The number of toilet seats in DCCs ranged from 1 to 12 seats with a mean of 4.3 ± 2.1 seats and the number of children per toilet seat ranged from 3 to 65 children with a mean of 12.5 ± 10.1 children.

Regarding the availability of infection prevention and control resources, about two thirds of DCCs (61.1%) did not have a room for medical examination. Stool analysis of the registered child was required in 69.5% of them. Almost all (98.3%) DCCs were well aerated, 94.9% were cleaned using disinfectant preparations (mostly chlorine solution) and 96.6% had a refrigerator for preserving the children’s meals. The bathrooms were cleaned more than once daily in 83.1% of the DCCs.

Alcohol-based products and gloves were present in less than half of the DCCs (44.1% and 45.8% respectively), while aprons were only available in 20.3% of DCCs. There was no diaper changing room in 8.4% of the DCCs, and in the remaining DCCs, half of them had separate rooms (designed only for changing diapers) and the other half had common rooms. Most of the diapers changing rooms (79.6%) were cleaned more than once daily, 55.6% contained hand washing facilities and in 44.4% gloves were available inside the room.

The mean age of caregivers was 31.6 ± 8.02 years, all of them were females, 52% were married while 42.8% were single, 60.7% were teachers while 37.6% were nannies, 59.5% had a university education, and 20.1% had basic education. The years of experience ranged from 2 months to 17 years with a mean of 2.9 ± 2.3 years. Only 12.4% of caregivers attended special training on child care, while almost all the caregivers (98.8%) did not attend any training on infection control.

**Fig. 1:**
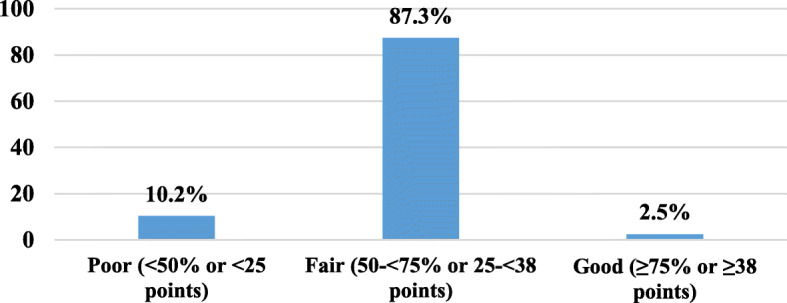
Level of knowledge of caregivers regarding infectious diseases and infection control measures (Alexandria, 2015)

Figure 1 illustrates that only 2.5% of caregivers had a good level of knowledge regarding infectious diseases and infection prevention and control measures, while 87.3% had a fair level of knowledge, and 10.2% had a poor level of knowledge. The mean knowledge score was 29.2 ± 4.3 points and ranged from 16 to 44 points.

Studying the effects of the personal characteristics of caregivers on their knowledge regarding infectious diseases and infection control measures, one-way MANOVA indicated only a statistically significant difference between caregivers with different levels of education on the combined dependent variables (infectious diseases and infection control mean knowledge score): *F* = 3.788, *p* = 0.005, Wilks’ lambda = 0.958, and partial Eta squared = 0.021, Table 1.

**Table 1:** MANOVA general *F*-test, factors affecting infectious diseases and infection control knowledge of caregivers (Alexandria, 2015)

Variable	Wilks’ lambda	*F*	*p* value	Partial eta squared	Observed power
Age	0.995	0.798	0.451	0.005	0.186
Experience years	0.998	0.464	0.629	0.002	0.126
District	0.980	1.768	0.133	0.010	0.542
Education	0.958	3.788	0.005	0.021	0.892
Child care training	0.992	1.348	0.261	0.008	0.290
Infection control training	0.996	0.695	0.500	0.004	0.167
Job	0.990	0.613	0.720	0.005	0.247
Marital status	0.993	0.613	0.653	0.004	0.203

Given the significance of the overall test, the univariate main effects were examined (Table 2). Significant univariate main effect for education was statistically associated only with infectious diseases knowledge after using a Bonferroni adjusted alpha level of 0.025 (*F* = 6.545, *p* = 0.002). Table 3 shows that caregivers with university education had a higher significant mean knowledge score about infectious diseases compared to those with other educational categories as revealed by the post hoc test results.

**Table 2:** MANOVA univariate test of factors affecting infectious disease and infection control knowledge of caregivers (Alexandria, 2015)

Variable	Knowledge	*F*	*p* value	Partial eta squared
Age	Infectious diseases	1.505	0.221	0.004
	Infection control	0.261	0.610	0.001
Years of experience	Infectious diseases	0.545	0.461	0.001
	Infection control	0.224	0.636	0.001
District	Infectious diseases	1.552	0.213	0.009
	Infection control	2.585	0.077	0.015
Level of education	Infectious diseases	6.545	0.002	0.036
	Infection control	0.731	0.482	0.004
Child care training	Infectious diseases	0.192	0.662	0.001
	Infection control	2.216	0.138	0.006
Infection control training	Infectious diseases	1.308	0.254	0.004
	Infection control	0.009	0.922	0.000
Job	Infectious diseases	0.909	0.437	0.008
	Infection control	0.163	0.921	0.001
Marital status	Infectious diseases	0.310	0.733	0.002
	Infection control	1.033	0.357	0.006

**Table 3:** Post hoc test using Tukey HSD for multiple comparisons of education as a factor affecting infectious disease knowledge among caregivers (Alexandria, 2015)

Variable	Education	Mean	Standard deviation
Infectious diseases knowledge	Below secondary	54.37^a^	12.635
	Secondary	53.53^b^	10.930
	University	64.19^ab^	13.307

^a^*p* = 0.000

^b^*p* = 0.000

Table 4 shows the results of multiple linear regression of factors affecting knowledge score of caregivers regarding infectious diseases and infection control. Level of education was the only significant predictor.

**Table 4:** Results of multiple linear regression of studied factors on caregivers’ knowledge score regarding infectious diseases and infection control (Alexandria, 2015)

Independent variable	Parameterestimate	*p* value	Standardizedestimate	95% confidence limits	
Age	0.07593	0.3743	0.07102	− 0.09190	0.24376
Years of experience	− 0.10362	0.6235	− 0.02794	− 0.51833	0.31109
Level of education Below secondary vs. university	8.22774	0.0136*	− 0.41776	− 14.75402	− 1.70146
Secondary vs. university	6.64894	0.0321*	− 0.27834	− 12.72499	− 0.57290
Teacher vs. other jobs	− 0.45370	0.8895	− 0.02586	− 6.86924	5.96185

*Significant (*p* < 0.05)

**Fig. 2:**
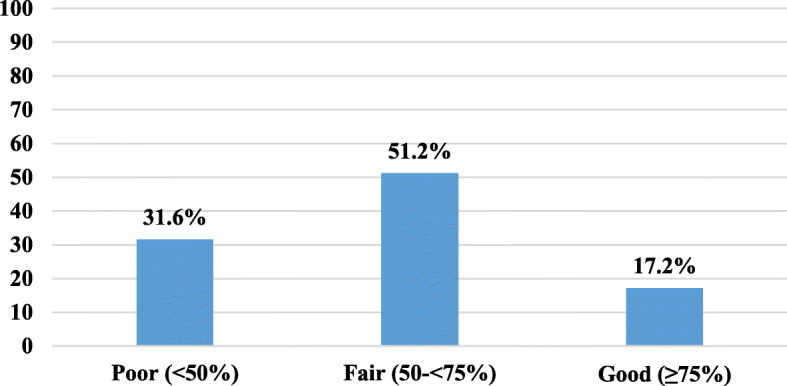
Distribution of infection control observations of caregivers according to the practice score percent (Alexandria, 2015)

Regarding the observation of infection control practices, Fig. 2 illustrates that in 51.2% of the observations, caregivers had fair score percent, while observations with poor and good score percent represented 31.6% and 17.2% respectively. The mean practice score percent was 58.2% ± 14.8% and ranged from 19.3% to 89.6%.

Table 5 shows that only two variables were significantly affecting the DCC caregivers’ infection control practice (poor = 0/fair and good = 1). The first was being from district X. Caregivers with good practice score were 2.5 times more likely to have been from district X compared to those with a poor score. The second was the working hours/day. Caregivers with good practice were 2.2 times more likely to be working more than 7 h/day compared to those with poor score. The model correctly classified 72.1% of the cases.

**Table 5:** Predictors of infection control practices of caregivers in DCCs (Alexandria, 2015)

Independent variables	Odds ratio	*p* value	95% Waldconfidence interval	
District X vs. Y	2.514	0.0007*	1.474	4.287
District Z vs. Y	1.598	0.1540	0.839	3.043
Teacher vs. other jobs	0.708	0.1728	0.430	1.163
Daily working hours	2.246	0.0010*	1.387	3.637
Distribution of children	0.878	0.4354	0.633	1.218
No. of children the caregiver is responsible for	0.985	0.1251	0.967	1.004

*Significant (*p* < 0.05)

Sensitivity of the model = 72.1%

## Discussion

Infection prevention and control form an important part of care provided in DCCs for children. DCCs’ attendance has always been an important factor in contracting diseases among children. As more and more children become enrolled in DCCs nowadays, attention should be paid toward providing infection-free care [15].

Guidelines for planning and designing quality child care facilities in Australia recommend that an education and care service premises must provide for every child being educated and cared for within the center to have a minimum of 3.25 m^2^ of unencumbered indoor space [19, 20]. It was noticed in the current study that the classes were relatively not overcrowded. Even in small DCCs, the number of children enrolled in them was small. The present study showed that the number of classes per DCC ranged from 2 to 7 with a median of 3 classes. The number of children per DCC class ranged from 1 to 23 with a median of 11 children per class, which was comparable to the findings of a study conducted in the Netherlands (2013) [4]. The current study recorded an availability of alcohol-based products and gloves in 44.1% and 45.8% of the DCCs which was much less than that observed in the Dutch study [4].

Guidelines from Australia recommend a minimum of 2 pans and 2 washbasins for a maximum of 30 children (with a ratio of 1 in 15), with an additional pan/washbasin required for each additional group of 15 children [20, 21]. The current results were in accordance with those findings, where the mean number of children per toilet seat was 12.5 ± 10.1 children.

Only 2.5% of caregivers in the present study had good knowledge about infectious diseases, their modes of transmission, and infection control practices, 10.2% had poor level of knowledge, and 87.3% had fair knowledge. Higher figures were reported from another study conducted in the Netherlands (2013) regarding knowledge of caregivers about hand hygiene guidelines [22]. The low level of knowledge of caregivers in the present study may have several reasons. First, the different educational backgrounds of caregivers. Second, they may consider that their level of personal hygiene is satisfactory and accordingly they did not perceive the need to acquire additional knowledge, although 59.5% of them were university graduates. Moreover, there were no specific requirements as regards medical knowledge for the employment in the DCCs whether as a teacher or a nanny. Another explanation pointed to by caregivers was the lack of guidelines in the DCCs themselves. Provision of administrative work regulations (like when to come and when to leave) was of more priority than the provision of infection control guidelines.

In the current study, the results of multiple linear regression showed that only the level of education was the significant predictor for the knowledge score. This may be explained by the fact that those who graduated from the university tend to be more knowledgeable compared to those with lower educational levels.

There was a gap between the infection control knowledge and practice of caregivers in the current study. Caregivers with a fair level of knowledge represented 87.3% while those with a fair level of practice only represented 48.5% denoting that they did not apply all what they knew. Most caregivers claimed to know the correct hand washing technique and reported that hands should be washed before meals, while during observation, much lower percent performed hand washing in the right way or washed their hands before meals. Poor training of caregivers (only 12.4% of caregivers had previous training on childcare), the high workload, and their relatively little payment that resulted in their high turnover could be possible explanations for these findings. On the other hand, only 2.5% of caregivers had good level of knowledge, while 20.9% had good level of practice. Most probably, those with good practice seemed to adopt good hygienic measures reflected on their behavior, regardless of their background.

Logistic regression analysis showed that only two variables were significantly affecting the practice. The first was being in district X. This might be due to the characteristics of district X, as the residents are of a higher socio-economic class than other districts. During the study, it was noticed that the monthly fees paid per child in DCCs in district X were double to triple those paid in DCCs from other districts. The second factor was the working hours per day. Those who worked for 7 or more hours per day may be more experienced than those who worked less than 7 h per day.

The results obtained from the present study were nearly similar to the baseline data of several interventional studies conducted to evaluate the effect of infection control programs in reducing infectious diseases among children attending DCCs. These programs were based on the fact that caregivers’ knowledge about infectious diseases, their modes of transmission, and infection control practices were marginal. After the implementation of these programs (mainly health education programs about hand hygiene and environmental cleaning), the infectious diseases rates among children and caregivers were reduced, enhancing the fact that level of knowledge and infection control practices among those caregivers was really poor [23, 24].

### Limitations of the study

National guidelines for designing DCCs, staff requirements, and the required infection control logistics and practices are not available and thus judging the current status in DCCs was difficult. Also, most of the available international guidelines lacked such details.

## Conclusion

DCC features were satisfactory regarding their aeration, level of cleanliness, and availability of hand washing facilities, while the availability of gloves and aprons, alcohol-based products, and medical examination rooms were not satisfactory. The level of knowledge and practice of caregivers regarding infectious diseases and infection control measures were below optimum. The level of education was the only significant predictor for the knowledge score, while being in district X and the working hours per day were the two variables significantly affecting the practice.

## Recommendations

Raising awareness by ongoing education in addition to regular training of caregivers about infectious diseases and infection control practices are recommended. All workers in DCCs should be certified in child care and related infection control practices. A medical examination room with a working physician is needed in all DCCs. Provision of national guidelines and regular monitoring and supervision of DCCs, particularly those with a large number of children is required.
